# Optimizing neuroscience data management by combining REDCap, BIDS and SQLite: a case study in Deep Brain Stimulation

**DOI:** 10.3389/fninf.2024.1435971

**Published:** 2024-09-05

**Authors:** Marc Stawiski, Vittoria Bucciarelli, Dorian Vogel, Simone Hemm

**Affiliations:** Neuroengineering Group, Institute for Medical Engineering and Medical Informatics, School of Life Sciences, FHNW University of Applied Sciences and Arts Northwestern Switzerland, Muttenz, Switzerland

**Keywords:** Neuroscience data, data management, Brain Imaging Data Structure (BIDS), Electronic Data Capture (EDC), Deep Brain Stimulation (DBS)

## Abstract

Neuroscience studies entail the generation of massive collections of heterogeneous data (e.g. demographics, clinical records, medical images). Integration and analysis of such data in research centers is pivotal for elucidating disease mechanisms and improving clinical outcomes. However, data collection in clinics often relies on non-standardized methods, such as paper-based documentation. Moreover, diverse data types are collected in different departments hindering efficient data organization, secure sharing and compliance to the FAIR (Findable, Accessible, Interoperable, Reusable) principles. Henceforth, in this manuscript we present a specialized data management system designed to enhance research workflows in Deep Brain Stimulation (DBS), a state-of-the-art neurosurgical procedure employed to treat symptoms of movement and psychiatric disorders. The system leverages REDCap to promote accurate data capture in hospital settings and secure sharing with research institutes, Brain Imaging Data Structure (BIDS) as image storing standard and a DBS-specific SQLite database as comprehensive data store and unified interface to all data types. A self-developed Python tool automates the data flow between these three components, ensuring their full interoperability. The proposed framework has already been successfully employed for capturing and analyzing data of 107 patients from 2 medical institutions. It effectively addresses the challenges of managing, sharing and retrieving diverse data types, fostering advancements in data quality, organization, analysis, and collaboration among medical and research institutions.

## 1 Introduction

Over the past few years, neuroscience research has undergone a significant transformation. Technological advancements have enabled acquisition of extensive, multi-modal datasets whose analysis can elucidate mechanisms underlying neurological disorders and unveil new treatments (Dipietro et al., 2023). However, extracting meaningful insights from neuroscience data is not a trivial problem. A first challenge is posed by dealing with a multifaceted data landscape (Dash et al., 2019). Neuroscience studies entail, in fact, the generation of heterogeneous data ranging from neuroimages to clinical evaluations, to demographics. The integration of diverse data types plays a crucial role in deriving clinically relevant conclusions (Sejnowski et al., 2014). Moreover, such data is sourced from different clinical institutions and is often gathered at various time intervals to facilitate longitudinal studies (Dipietro et al., 2023). Data collection within hospital settings often relies on traditional methods such as paper-based documentation or static spreadsheets (Poline et al., 2012; Saczynski et al., 2013). These methods, despite being straightforward and widespread in clinics, may present some limitations in data standardization and accessibility (Wilcox et al., 2012). After collection, data needs to be transferred to research institutes to perform further analyses. According to data sharing best practices (Ferguson et al., 2014), data should follow the FAIR (Findable, Accessible, Interoperable, Reusable) principles (Wilkinson et al., 2016). However, sharing data recorded using the above-mentioned methods for collaborative research purposes, raises concerns about data security and integrity. Therefore, systematic data management is imperative to leverage the insights derived from neuroscience multi-modal data. This can be achieved by employing systems facilitating efficient data capture, organization, management, and secure sharing (DiEuliis and Giordano, 2016; Dash et al., 2019; Dipietro et al., 2023).

In the last years several Electronic Data Capture (EDC) systems, such as REDCap (Harris et al., 2009), CARAT (Turner et al., 2011) and CIGAL (Voyvodic et al., 2011), have emerged to address the limitations of paper-based and spreadsheet-based data collection. These systems offer web-based interfaces for multi-modal data capture and can also provide data verification functions (Turner and Van Horn, 2012; Li and Liang, 2022). However, EDC systems do not support image files (Deserno et al., 2013). Medical images are usually stored in specialized Picture Archiving and Communication Systems (PACS) (Choplin et al., 1992). While PACS offer secure storage and retrieval of medical images, they are not designed for integrating clinical data with imaging data (Scott et al., 2011). Furthermore, lack of standardization in file structures and naming, severely hinders efficient data management in analysis pipelines, data sharing and study reproducibility (Zwiers et al., 2022). This issue has been tackled by introducing data storage specifications such as Experimental Directory Structure (ExDir) (Dragly et al., 2018) or Brain Imaging Data Structure (BIDS) (Gorgolewski et al., 2016). The latter is a standard describing how to organize neuroimaging data and related metadata following a specific folder structure and naming convention. BIDS was initially designed to accommodate raw imaging data. Guidelines to store analysis-derived data have been recently introduced but they remain quite broad and may not encompass all file types generated in the various neuroscience fields. Given its growing usage in the neuroimaging community, the last years have witnessed the development of tools providing semi-automated organization of imaging datasets according to BIDS guidelines (Lopez-Novoa et al., 2019; Zwiers et al., 2022; Levitas et al., 2024) or bridging between BIDS datasets and software solutions (Jegou et al., 2022). In order to exploit the joint power of imaging and clinical data, a recent work has proposed a solution integrating data collected through REDCap with imaging data organized according to BIDS (Kuplicki et al., 2021). However, it lacks the ability to present a comprehensive data overview. Addressing this last point, several extensive data management and sharing systems have been developed. They have adopted diverse strategies to provide functionalities such as data capture, storage, sharing, import, export, processing, retrieval, quality control, provenance tracking (Marcus et al., 2007; Keator et al., 2009; Van Horn and Toga, 2009; Prodanov, 2011; Scott et al., 2011; Das et al., 2012; Book et al., 2013; Muehlboeck et al., 2014; Woodman et al., 2014; Grigis et al., 2017). Nevertheless, they may entail considerable load related to installation, maintenance, and user training (Kuplicki et al., 2021). Moreover, there is no universal solution fitting all the possible use-cases arising in the neuroscience field (Van Horn and Toga, 2009). To achieve the maximum possible efficiency, a data management system should be tailored to the workflows generating and using the data stored in it.

Therefore, this manuscript presents a data management system providing solutions to handle extensive, heterogeneous data and designed to support Deep Brain Stimulation (DBS) research pipelines. DBS is a widely employed neurosurgical procedure for managing symptoms of a variety of movement and psychiatric disorders (Hariz et al., 2013; Johnson et al., 2013; Wårdell et al., 2022). During the whole DBS process huge amounts of heterogeneous data are generated. Group analysis (e.g., analysis of data collected from multiple patients) is crucial in enhancing understanding of the relationship between stimulation parameters and clinical outcomes (Neudorfer et al., 2021; Nordin et al., 2022). With the aim of enhancing the efficiency and reproducibility of DBS research, the system described in this paper leverages the integration of REDCap, BIDS and SQLite.^1^ It offers the following functionalities:

(1) Straightforward data capture in hospital settings
(2) Secure data sharing between clinics and research institutes
(3) Management of clinical and imaging data types
(4) Standard-based imaging data organization
(5) Linkage of image files and related metadata
(6) Access to heterogeneous data through a unique tool

## 2 System design and workflows

This section describes data acquired during a typical DBS procedure and the methodological framework used to create a data management system for DBS research. The presented comprehensive data management system combines existing data aggregation platforms and standards, with custom software for data handling. It utilizes REDCap as EDC system, BIDS as image storing standard, and an SQLite database as a comprehensive data store. Custom Python scripts connect the different components, ensuring efficient platform interoperability.

### 2.1 DBS procedure and data

#### 2.1.1 Raw data acquisition

DBS entails the implantation of electrodes in specific deep brain targets (e.g. Subthalamic Nucleus, Zona Incerta), whose electrical stimulation leads to disease symptom reduction (Lozano et al., 2019). The DBS procedure varies between medical centers but can, in general, be split into three main phases: pre-operative planning, implantation and post-operative follow-up (Hemm and Wårdell, 2010; Shah et al., 2020). All three phases include the generation and acquisition of heterogeneous types of patient data. Pre-operative planning consists in delineating the precise brain target and trajectory to reach it, based on pre-operative anatomical images (D’Haese et al., 2005; Rezai et al., 2006). Patient demographics, medical history and anatomical imaging (e.g. CT or MRI) data, electrode type and targeting information are therefore collected in this first phase. During surgery electrophysiology techniques, such as microelectrode recording (MER) and intra-operative stimulation tests are often applied to identify the optimal electrode position (Schrader et al., 2002; Miyagi et al., 2009). The DBS leads are inserted afterwards in the brain for chronic stimulation. Post-operatively the final electrode position is checked through CT or MRI imaging (Chabardes et al., 2015). DBS parameters (e.g. active contact, current amplitude, pulse width) are tuned in a series of consultations which involve symptom evaluation through disease-specific clinical scoring scales (Koeglsperger et al., 2019; Wårdell et al., 2022). Outcomes of intra-operative and post-operative stimulation tests are recorded in paper forms.

#### 2.1.2 Data analysis

The data acquired during the DBS procedure constitutes the input to analysis workflows, which in turn create additional data streams. In particular, the generation of electrodes, electrode trajectories and brain tissue models (brain conductivity matrix) allows to simulate the Volume of Tissue Activated (VTA) by specific stimulation configurations (Butson et al., 2006; Åström et al., 2015; Alonso et al., 2016; Horn et al., 2019; Latorre and Wårdell, 2019; Butenko et al., 2020; Neudorfer et al., 2023). VTAs of multiple patients can be jointly examined in a common anatomical space, also called anatomical template or atlas, obtained by fusing together preprocessed images of several patients (Evans et al., 1994; Lemaire et al., 2010; Vogel et al., 2020). Sometimes additional anatomical information such as manually outlined structures (Lemaire et al., 2010) can be projected and added to the template. Statistical approaches applied to patient data in a common reference space allow to extract probabilistic stimulation areas (Butson et al., 2011; Eisenstein et al., 2014; Reich et al., 2019; Nordin et al., 2022). These can in turn be used to learn more about the therapeutic targets or they can be transformed to the image space of new patients and used to support in determining their optimal stimulation parameters (Roediger et al., 2021; Nordenström et al., 2022).

#### 2.1.3 Data type categories

DBS data from two medical institutions (University Hospitals of Basel and Clermont-Ferrand) was collected and analyzed to validate the system presented in this manuscript. The data types handled by the system were classified into 2 macro-categories. The first includes raw data acquired during the DBS procedure, which can be further subdivided into clinical and imaging data. The second category encompasses all the data created by the analysis workflows.

• Raw data:
◦ Clinical data: detailed patient demographics, medical history, medication schedules, surgical details (e.g. targeting and final implanted position information), stimulation parameters, symptoms scoring.
◦ Imaging data: pre and post-operative anatomical images (e.g. CT or MRI scans), labeled anatomical structures.

• Analysis data: preprocessed anatomical images (e.g. skull-stripped), reconstructed electrode trajectories, electrode COMSOL^2^ models, brain conductivity matrix, simulated VTAs, anatomical atlas images, segmented anatomical structures, probabilistic stimulation maps, images transformed from patient reference space to atlas reference space and vice-versa, transforms and warps needed to perform image transformations.

**FIGURE 1:**
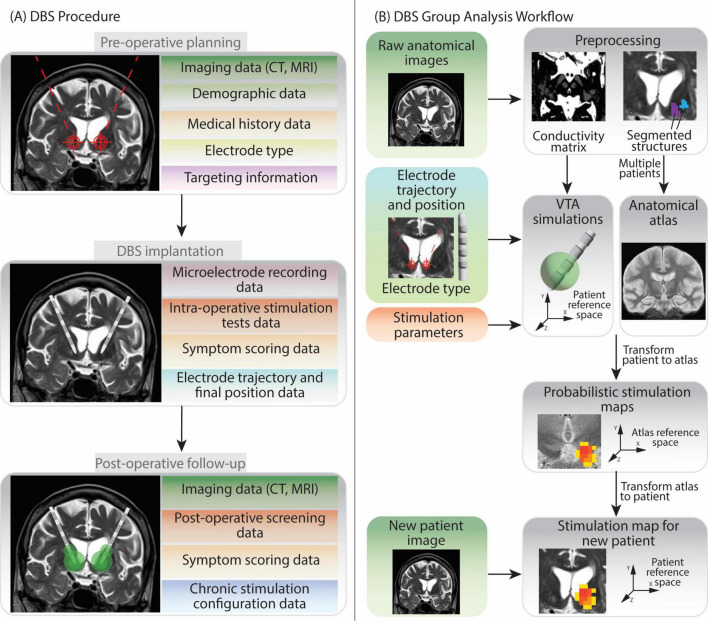
Overview of Deep Brain Stimulation procedure and group analysis workflow. The figure shows the various types of data generated at each step, in light of the DBS protocols followed by the two medical centers contributing to the data collection in this work. **(A)** The DBS procedure can be split in three phases: pre-operative planning, implantation and post-operative follow-up. Data generated during pre-operative planning encompasses patients’ demographic information, medical history, anatomical brain images, the model of DBS electrode chosen for implantation and the planned target point and trajectory. During implantation recording of brain cells activity (microelectrode recording) and stimulation tests help to determine the final, optimal electrode position. In the post-operative follow-up additional anatomical imaging data and stimulation parameters evaluations (screening) data are collected. Moreover, information on the final chosen chronic stimulation configuration is also recorded. **(B)** The group analysis workflow firstly requires patients’ anatomical images preprocessing. Subsequently Volume of Tissue Activated (VTA) simulations can be run for each patient and each stimulation configuration. Several patients’ anatomical images can be fused together to create a common anatomical space (anatomical atlas) (Vogel et al., 2020). Patients VTAs are then transformed into the atlas reference space so that they can be superimposed and analyzed together to generate Probabilistic Stimulation Maps. These maps indicate brain areas leading to high symptom improvement when stimulated. When data from a new patient is acquired, the stimulation map can be transformed to their reference space. The data elements in the left part of image **(B)** are collected during the DBS procedure, while the ones on the right (gray background) are generated during the data analysis.

The main data types collected during the DBS procedure by the medical centers contributing to this work, are summarized in Figure 1A. The group analysis workflow schema is represented in Figure 1B.

### 2.2 Data management system use case analysis

A user-centered design approach was employed to define system requirements. This involved identifying the various user roles and their corresponding use cases. By analyzing these use cases functional requirements were derived. These outline the specific actions the system should be able to perform. In a second step, non-functional requirements were added, addressing the system’s characteristics such as security, performance, and scalability. The system caters to two primary user groups: clinicians and researchers, each with distinct use cases.

• Clinician
◦ Use Case 1.1: Easy Data Entry: clinicians can effortlessly record patient information through user-friendly, structured forms within the web interface. This minimizes the data entry burden and promotes accuracy.
◦ Use Case 1.2: Data Export for Statistical Analysis: clinicians can export collected data, either for a single patient or an entire cohort. Data can be exported in a format suitable for statistical analysis using a software of choice.
◦ Use Case 1.3: Data Edit: clinicians can edit data or fill in incomplete records.

• Researcher
◦ Use Case 2.1: Data Retrieval: researchers can access clinician-captured data. They can filter the data according to specific metadata to narrow their analyses to specific patient groups.
◦ Use case 2.2: Data Edit: researchers can correct or update data.
◦ Use case 2.3: Data Analysis: researchers can apply processing steps to retrieve data, including image processing and clinical data analysis.
◦ Use case 2.4: Results Saving and Sharing: researchers can integrate generated results back into the system and make them available to other users.

**FIGURE 2:**
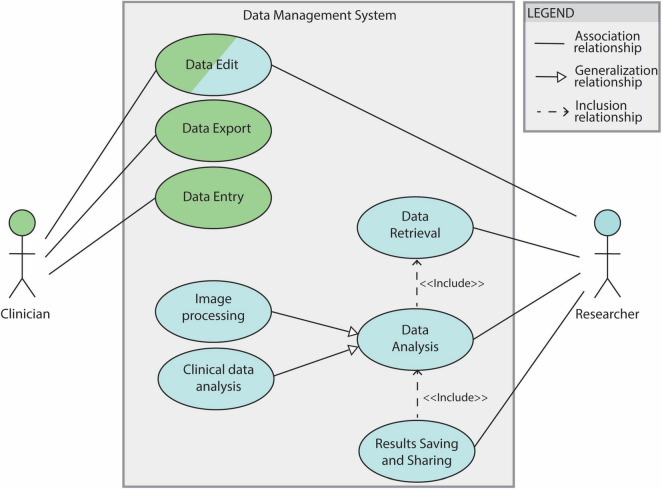
Use case diagram for the DBS data management system. The system features clinicians and researchers as actors. Filled lines without arrow indicate the association between the actor and the use case. Filled lines with white-filled arrow point indicate that a use case is generalization of another. In this diagram “Image processing” and “Clinical data analysis” are 2 specifications of the general use case ‘Data analysis’. The dotted lines indicate ‘Include’ relationships between use cases: in order to be able to save and share results a data analysis needs to be performed (’Data Analysis’ is therefore included by the “Results Saving and Sharing” use case). The “Data Analysis” use case depends, in turn, on the “Data Retrieval” one.

Use cases 2.1, 2.3 and 2.4 are fundamental steps of the DBS group analysis workflow (see section 2.1.2). The overall use case diagram of the data management system is shown in Figure 2.

### 2.3 System components

REDCap (Harris et al., 2009) was chosen as secure EDC web platform to streamline patient-related clinical data. Its user-friendly interface allows to create structured forms with data validation, and role-based access control (Supplementary Figures 1–6). REDCap serves as a centralized repository, simplifying retrieval and analysis. It offers real-time data capture, accessibility, and built-in reporting tools for a better understanding of patient cohorts and treatment outcomes (Server info: REDCap 14.3.2 PHP 7.4.33 (Linux/Unix OS) MariaDB 10.5.23).

BIDS (Gorgolewski et al., 2016) was chosen as imaging data storing standard. It acts as a common language for organizing brain imaging data, including MRI scans which are vital for DBS research. This standardized format ensures consistency in file naming conventions and folder structures across studies, streamlining collaboration and data sharing (used version: v1.9.0).

SQLite empowers research with its lightweight design. This self-contained database eliminates server needs and simplifies deployment, allowing researchers to focus on their work. Furthermore, SQLite’s SQL compatibility ensures familiar data management through a proven language (used version: 3.45.2).

Python excels as a data handling tool for research thanks to its extensive libraries like Pandas and NumPy, which enable efficient manipulation and analysis of complex datasets. The open-source nature of Python’s data engineering ecosystem fosters collaboration and facilitates the sharing of code and libraries for data handling tasks.

These components, except for REDCap, are designed for portability, meaning they can be run on any operating system without modification. This independence allows them to be easily rebuilt and deployed across different environments.

### 2.4 Clinical data management

**FIGURE 3:**
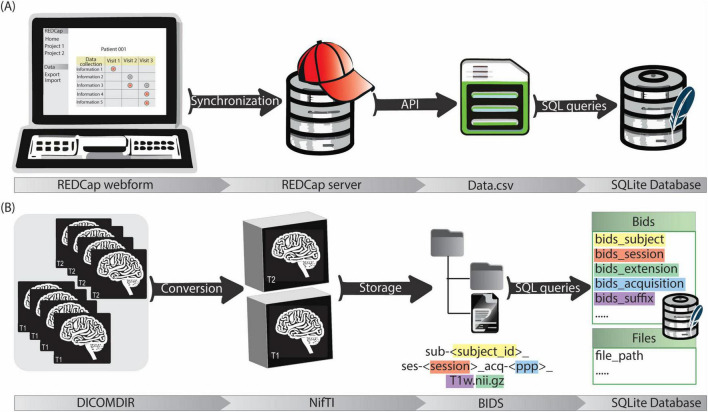
**(A)** The clinical data management workflow starts with the filling of customized REDCap webforms. The data is then synchronized with REDCap server for secure storage. Data is exported in.csv format via API and then stored in the SQLite database by a python script. **(B)** Imaging data is firstly acquired in DICOMDIR format and converted in NifTI format by a custom script. NifTI files are then named according to BIDS and stored in a repository with a BIDS-compliant structure. Image metadata (e.g., session, acquisition, suffix…) and file paths are saved in the SQLite database by a python script.

For each participating center a REDCap project was established in the REDCap instance, incorporating all relevant user roles and access rights. Customized forms were designed to best capture data originating from diverse sources. They encompass patient information, intra-operative details (e.g. targeting information, microelectrode recordings, implanted electrode position), and post-operative data including chronic stimulation settings. The acquired data is automatically stored on the REDCap server. To facilitate data retrieval and management, a software pipeline was developed in Python^3^. This pipeline utilizes REDCap’s API with appropriate tokens to automatically pull data from the server. The retrieved data undergoes a transformation process based on pre-defined mapping files, using libraries like regular expressions (regex) for pattern matching and data cleaning, and Pandas for data handling. Finally, SQL queries are generated and employed to insert the transformed data into the SQLite database (Figure 3A). The entire process, from database creation (if necessary) to data retrieval, cleaning, and import, is encapsulated within the software pipeline. The generic nature of the code allows its application across all REDCap projects.

### 2.5 Imaging data management

A dedicated data processing pipeline integrates the imaging data into the data management system. DICOM^4^ data from the neurosurgical planning station of participating centers is anonymized and exported in DICOMDIR format. This DICOMDIRs includes pre- and post-operative CT scans, as well as various MRIs with some labels and trajectories. The pipeline sets up a root directory for the image data storage. Then it utilizes a pre-built DICOM to Neuroimaging Informatics Technology Initiative (NIfTI) format converter. The converter automatically extracts information from the DICOMDIRs, converts the DICOM data into the NIfTI format, and stores the converted files according to the BIDS standard. Finally, a self-developed Python tool (Image2BIDS2SQLite^5^) generates image references through SQL queries and loads them into the SQLite database (Figure 3B).

**FIGURE 4:**
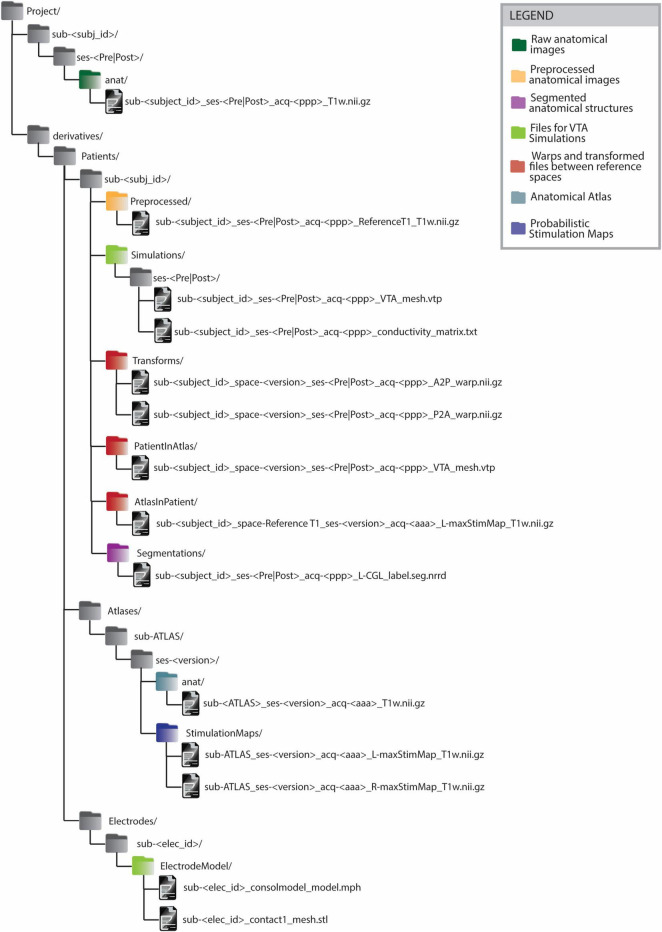
BIDS-compliant folder structure for Deep Brain Stimulation imaging files and processing outputs. All the files generated from some processing or analysis step are saved as BIDS ‘derivatives’. The derivatives files are grouped firstly by subject type (’Patient’, ‘Atlas’, ‘Electrode’) and on a second level by subject ID. The naming convention of files in folders such as ‘PatientInAtlas’ or ‘AtlasInPatient’ allows to keep track of transformations between different image spaces. Anatomical atlas images are saved as belonging to an ATLAS subject. Anatomical atlases generated from different patient groups or from data acquired with different imaging modalities are distinguished by the ‘version’ parameter. The legend allows identifying the groups of files generated or used in the different steps of group analysis in Deep Brain Stimulation.

The BIDS-compliant data structure (Figure 4) features a division between raw and analysis data (see section 2.1.3). Data differing from raw anatomical images is, in fact, saved within dedicated ‘derivatives’ folders. Inside the ‘derivatives’ folder, files are categorized according to the subject to which they refer. This classification is performed in 2 steps: firstly by distinguishing the type of subject (e.g. ‘Patient’, ‘Atlas’, ‘Electrode’) and then by indicating each subject with its unique subject ID. The subject type ‘Atlas’ includes the group analysis results contained in a common reference space. Since anatomical atlases are generated from multiple patients’ images, several atlases can exist. Thus, the ‘version’ distinguishes atlases generated from different data or image modalities. The ‘Electrode’ subject type includes the electrodes used for implantation. Each electrode is characterized by a different structure and therefore a different model to be used for electrical field simulations. Within the subjects’ subfolders, files are organized by content rather than by process. For instance, the folder ‘Segmentations’ contains segmented anatomical structures. Naming conventions such as the tag “PatientInAtlas” are used to facilitate tracking transformations between different imaging spaces. Such information is also captured in the ‘space’ field of the transformed image file. For instance, when transforming the file sub-<subject_id>_ses->session<_acq->ppp<_T1w.nii.gz to the image space of sub-ATLAS_ses-<version>, the output file will be named sub-<subject_id>_space-<version>_ses-<session>_acq-<ppp>_T1w.nii.gz and will be saved in the folder derivatives/sub-<subject_id>/PatientInAtlas. This structure essentially functions as a clear legend, allowing researchers to readily identify the data used and generated during various stages of the workflow.

### 2.6 Comprehensive data management

The system relies on SQLite, a relational database management system, to seamlessly integrate clinical data and image paths within a centralized system. This approach streamlines data organization and facilitates efficient retrieval of clinical information alongside corresponding image files.

**FIGURE 5:**
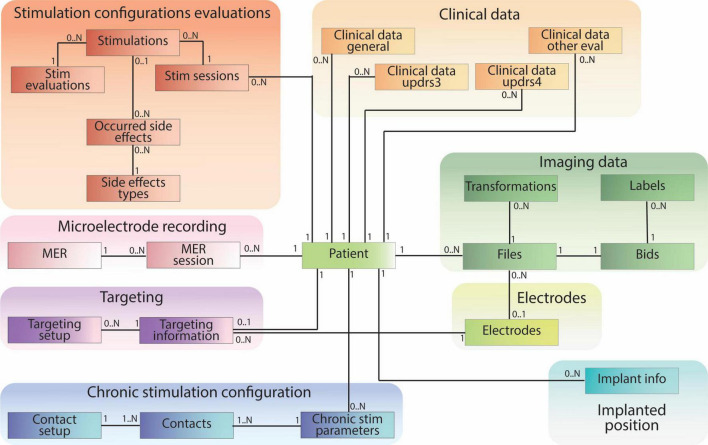
Patient-centric relational database schema, including clinical data pulled from REDCap and references to raw and processed imaging files stored according to BIDS. The database schema is designed mirroring the different data types collected during the DBS procedure.

The patient-centric database schema consists of 28 tables containing 230 data fields (e.g., patient ID, age, implant position, image file path) and 33 established relationships between these tables. These tables can be grouped into specific categories based on information content (e.g., Clinical Data, Imaging Data, Stimulation Configuration Evaluations, Targeting). Notably, all groups except for the Electrode table have a direct connection to the central patient table (Figure 5).

Clinical data tables encompass information collected during clinic visits. The “Clinical Data General” table stores medication data, while three other tables within this group capture data from relevant DBS clinical evaluations. Tables within the “Stimulation Configuration Evaluation” group store data regarding intra-operative or post-operative stimulation settings, their clinical effects, and potential side effects. “Microelectrode Recordings” tables specifically house data from intra-operative recordings used to identify distinct brain regions. The “Targeting” tables contain data relevant to pre-operative planning and arc settings. “Chronic Stimulation Configuration” stores information about patients’ post-operative electrode settings for daily use. The “Implanted Position” table captures the placement of the electrode after surgery. The “Electrodes” table stores data about the studies’ relevant electrodes and links to the “Files” table to establish a direct path to electrode-specific files.

**FIGURE 6:**
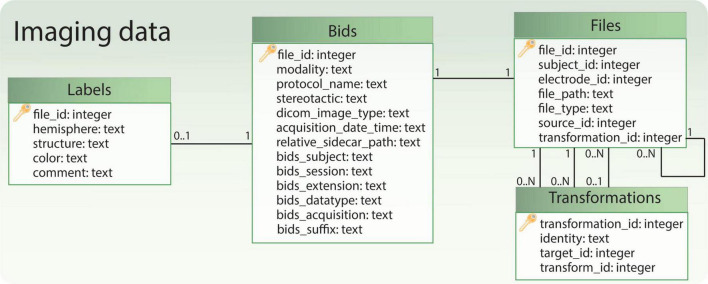
Relational database tables handling references to imaging files. The table ‘Bids’ contains some BIDS-specific image information, while the table ‘Files’ allows to retrieve the file path and the subject to which the files refer. Moreover, together with the table ‘Transformations’ it allows to keep track of image transformations between different spaces (e.g., patient, anatomical atlas). Finally, if the file represents a labeled anatomical structure, some additional information is captured in the table ‘Labels’.

Imaging data is managed through four tables (Figure 6). The “Files” table stores the relationship to the patient or electrode, the file path, and the general file type. If a file originates from another file, this information is documented in the “source_id” field, which links to the original file. The “transformation_id” field describes the relationship to the “Transformations” table, specifying the transform used to generate the file. The “Transformations” table stores the “transform_id” representing a transform (warp) file and the “target_id” representing the target image file (e.g. the reference image space of the transformation output). The remaining tables, “Bids” and “Labels,” share the same ID as the “Files” table. They store additional information specific to file types. For instance, the “Labels” table includes a field indicating the anatomical brain structure represented by the label file, while the “Bids” table contains BIDS-specific details for images, such as acquisition time and modality.

In conjunction with the database, a collection of Python functions was designed to facilitate data insertion, updating, deletion, and querying operations. This approach obviates the necessity for researchers to write intricate SQL queries while managing data for analysis.

## 3 System functionalities and practical application

This paper presents a data management system that leverages the strengths of REDCap, BIDS and SQLite to streamline data capture and organization in neuroscience based on an application example in DBS research. The system effectively addresses the challenges associated with managing diverse data types, including patient-related information, medical images, and post-processing results.

### 3.1 Data management system overview

The system performs three main tasks:

1. Clinical Data Management
2. Image Data Management
3. Comprehensive Data Management

**FIGURE 7:**
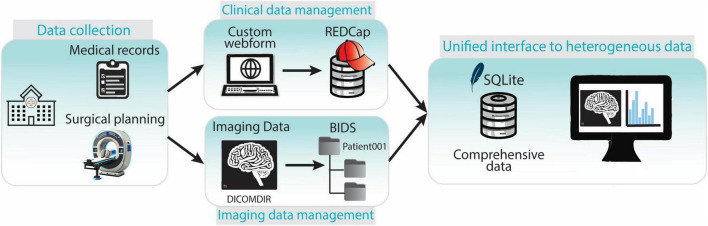
Overview of the components of the data collection and management framework. Clinical and imaging data is collected in the hospital. Clinical data is entered in the REDCap web-platform while imaging data is stored and transferred in DICOMDIR format. DICOMDIR data is converted in NifTI format and stored in a BIDS-compliant repository. References to medical images, images metadata and clinical data are stored in an SQLite database providing a unified interface to all the heterogeneous types of data.

These components work together seamlessly to ensure efficient data capture, organization, and retrieval (Figure 7).

The data management system addresses the requirements of both clinicians and researchers involved in DBS studies. Specifically, it enabled functionalities that were previously unattainable with earlier data collection and management techniques and enhanced the efficiency and automation of existing functions. This section provides a detailed overview of the new framework’s impact compared to the previously used methods, referring to the use case analysis of section 2.2 and the identified functional system requirements listed in Tables 1, 2. The system also addresses the non-functional requirements of data security and integrity, system efficiency, usability, accessibility and regulatory compliance. Table 3 presents a short description of such requirements and how they are fulfilled. In all three tables the requirements are identified by an alphanumeric code (e.g. F.x indicates functional ones, nF.x indicates non-functional ones).

**TABLE 1:** Clinician use cases: The table details the functional requirements of the developed data management system related to the clinician use cases.

Code	Requirement definition	Fulfilled		Comment
		Old	New	
**Use Case 1.1: Easy Data Entry**				
1.1-F.1	Record data on a patient-by-patient basis	Yes	Yes	REDCap’s web-based interface allows clinicians to enter patient data efficiently through structured forms.
1.1-F.2	Identify mandatory data fields	No	Yes	
1.1-F.3	Field-level data validation (e.g., data type restrictions)	No	Yes	
1.1-F.4	Record data as a batch, by importing already collected table-based data	-	Yes	
**Use Case 1.2: Data Export**				
1.2-F.5	Export whole data of entire cohorts	Yes	Yes	REDCap facilitates exporting relevant datasets (entire cohorts or specific records).
1.2-F.6	Export filtered data of defined patients	Yes (manual)	Yes (automated)	
1.2-F.7	Export data in different formats for statistical analysis	No	Yes	
**Use Case 1.3: Data Edit**				
1.3-F.8	Track incomplete form entries for easy follow-up	No	Yes	REDCap shows the state of the forms to enable clinicians to track their data entries.
1.3-F.9	Track changes in data fields	No	Yes	

Each requirement is identified by an alphanumeric code and is associated to a brief description. The table also shows whether and to what extent each requirement is fulfilled by the proposed system and whether it was fulfilled by the previously employed data management methodology.

**TABLE 2:** Researcher use cases: The table details the functional requirements of the developed data management system related to the researcher use cases.

Code	Requirement definition	Fulfilled		Comment
		Old	New	
**Use Case 2.1: Data Retrieval**				
2.1-F.10	Access clinician-captured data	Yes (manual)	Yes (Partially automated)	Clinical data can be transferred through REDCap API. The image data needs to be manually transferred.
2.1-F.11	Organize and store data with a well-defined granularity	No	Yes	The database schema allows the data to be stored in patient-specific granularity and queried with support functions.
2.1-F.12	Employ search queries	No	Yes	
2.1-F.13	Data provenance and transformations applied to data	No	Yes	Thanks to the database schema, it is possible to see the history of data and the applied transformations.
**Use Case 2.2: Data Edit**				
2.2-F.14	Editing existing data entries	Yes	Yes	The SQLite database functions allow to insert or update records.
**Use Case 2.3: Data Analysis**				
2.3-F.15	Interact seamlessly with external data processing tools for analysis workflows (data import and export)	Yes	Yes	The SQLite database can be easily accessed by processing pipelines.
**Use Case 2.4: Results Saving and Sharing**				
2.4-F.16	Share research findings with authorized users within the system	Partial	Partial	The analysis results are shared, but no reports of the individual users.
2.4-F.17	Save analysis output files according to a defined standard.	Yes (manual)	Yes (automated)	Analysis output data is named according to BIDS and saved in the BIDS ‘derivatives’ folder.

Each requirement is identified by an alphanumeric code and is associated to a brief description. The table also shows whether and to what extent each requirement is fulfilled by the proposed system and whether it was fulfilled by the previously employed data management methodology.

**TABLE 3:** The table details the non-functional requirements of the developed data management system. These requirements are more general and therefore apply to all actors.

Code	Requirement definition	Fulfilled		Comment
		Old	New	
**Non-Functional Requirements**				
3.0-nF.18	Data Security and Integrity: the system prioritizes robust security measures to safeguard data confidentiality and prevent unauthorized access. Data integrity is ensured through validation checks and version control mechanisms.	Partial	Yes	REDCap system which is accessible from the web fulfills these conditions and the rest of our database management system is only accessible within the personal network.
3.0-nF.19	System Efficiency: The framework is designed for optimal performance, minimizing data processing times and ensuring responsiveness for all users.	No	Yes	All parts of our data management system show outstanding efficiency, whether via the web using REDCap or directly with the SQLite database.
3.0-nF.20	User-Friendliness (Usability): Ease of use for users with varying technical backgrounds.	Partial	Partial	REDCap fulfills the conditions of good usability with its graphical user interface. However, the rest of the data management system is not equipped with a graphical user interface.
3.0-nF.21	Accessibility: users can access the system from any location with an internet connection, facilitating remote data entry and retrieval.	Yes	Yes	As a web server, REDCap is accessible to doctors entering data from anywhere. The rest of the data management system is only accessible locally for the research group.
3.0-nF.22	Regulatory compliance: The system adheres to relevant data privacy regulations such as GDPR (General Data Protection Regulation) and HIPAA (Health Insurance Portability and Accountability Act) to ensure patient data protection.	–	Yes	With REDCap and the rest of the data management system, the system fulfills all the necessary regulatory aspects.

Each requirement is identified by an alphanumeric code and is associated with a brief description. The table also shows whether and to what extent each requirement is fulfilled by the proposed system and whether it was fulfilled by the previously employed data management methodology.

### 3.2 Clinician requirements

REDCap’s web-based interface allows clinicians to enter patient data efficiently through structured forms satisfying all four requirements from use case 1.1 (Easy Data Entry). On the other hand, paper or spreadsheet-based data collection does not satisfy requirements 1.1-F.2 (Identify mandatory data fields) and 1.1-F.3 (Field-level data validation) and requirement 1.1-F.4 (Record data as batch) is completely not applicable for such methodologies. REDCap also facilitates exporting relevant datasets (either entire cohorts or specific records) to enable clinicians to perform further analyses, satisfying all three requirements from use case 1.2 (Data Export). Paper or spreadsheet-based data collection methodologies can satisfy requirement 1.2-F.5 (Export whole data of entire cohorts) by copying the files. They can also meet requirement 1.2-F.6 (Export filtered data of defined patients) but implying a substantial manual effort while requirement 1.2-F.7 (Export data in different formats for statistical analysis) cannot be fulfilled without the usage of an additional file converter. Moreover, paper and spreadsheet-based methodologies do not allow to track changed and missing values, not suiting the requirements of use case 1.3. At the same time, REDCap shows the up-to-date forms status, and tracks changes in each value field.

### 3.3 Researcher requirements

For researchers, accessing and re-formatting data collected in hospital settings can be a time-consuming and error prone task (Use Case 2.1: Data Retrieval). Thanks to REDCap’s secure API researchers can avoid having to manually transfer clinical data which is automatically streamed to the SQLite database. Therefore, requirement 2.1-F.10 (Access clinician-captured data) is fulfilled in a matter of minutes against the hours or days needed when extracting information from paper or Excel-based reports. Furthermore, the data format is homogeneous across all patients included in the same REDCap project. On the downside, the image data currently still needs to be manually moved. Following the data transfer, the SQLite database enables data storage in a self-defined data schema. The researcher can then query both clinical and imaging data with a specific granularity while being informed of data provenance and modifications. Requirements 2.1-F.11 (Organize and store data with a well-defined granularity for efficient management), 2.1-F.12 (Employ search queries to rapidly retrieve specific clinical or imaging data) and 2.1-F.13 (Be aware of data provenance and of the transformations applied to data) are seamlessly satisfied. Even the most well-organized file systems and spreadsheets would only partially fulfill these requirements. In addition, data retrieval would take tens of minutes to one hour depending on the dataset size and domain-expertise of the user, compared to the few minutes required to query the database independently from the user’s familiarity with the data.

Requirement 2.2-F.14 (Edit existing data entries for corrections or updates) is satisfied by both new and old frameworks. Nevertheless, the suite of Python functions available for inserting or updating database entries offers a unique method of interacting with the data. This approach eliminates the need to adapt the methodology based on varying data formats. Similar considerations can be made regarding requirements 2.3-F.15 (Interact seamlessly with external data processing tools for analysis workflows), 2.4-F.16 (Share research findings with authorized users within the system) and 2.4-F.17 (Save analysis output files according to a defined standard). In principle both the SQLite database and a simple file system can be accessed by processing pipelines and permit to save output files as desired. The strength of the proposed framework resides in the univocity of the data source interrogated by analysis workflows, relieving the user from needing to know each file’s specific location. Furthermore, saving the analysis results according to the BIDS standard, along with the automatic creation of references to these files in the database, prevents data duplication: a reduction of the total number of files by two-thirds was observed in the context of this work.

**FIGURE 8:**
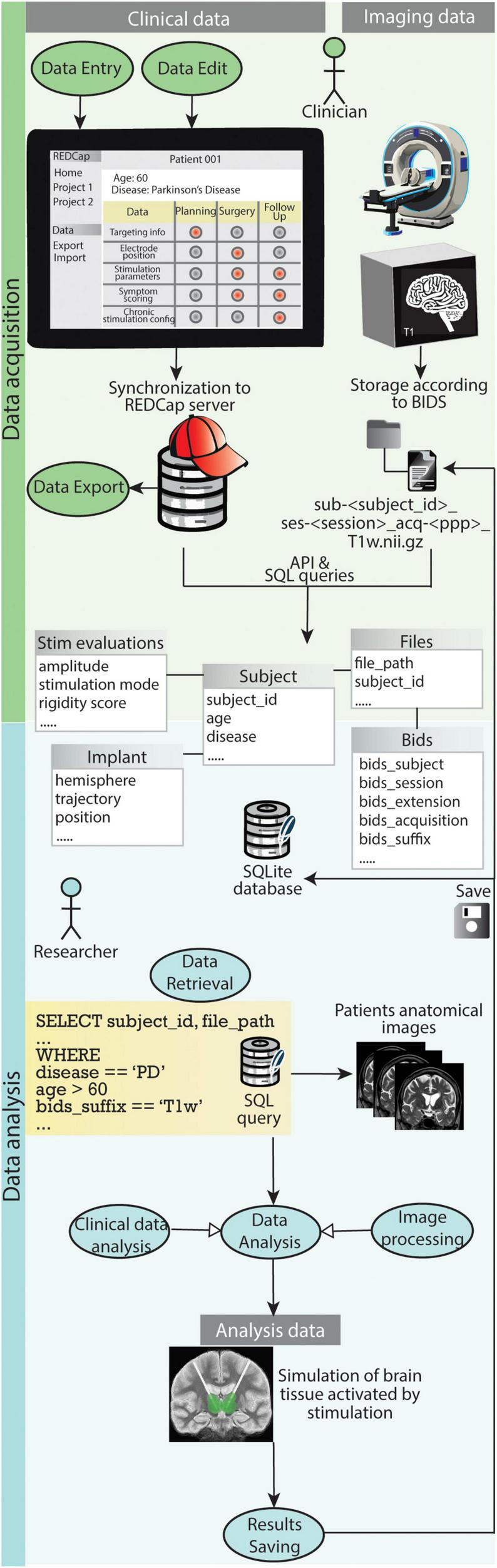
Graphical representation of how the use cases for clinicians and researchers play out with the support of the presented data management system. During data acquisition REDCap allows clinicians to easily carry out use case 1.1 (Data Entry), use case 1.2 (Data Export) and use case 1.3 (Data Edit). The file organization according to BIDS and storage of both clinical data and references to imaging files in the SQLite database enables researchers to efficiently retrieve specific data (use case 2.1). Such data is fed to processing pipelines for analysis (use case 2.3) and results are saved back into the database and the BIDS project folder (use case 2.4).

Figure 8 shows how the use cases for clinicians and researchers play out with the support of the presented data management system.

### 3.4 Application example

To validate the proposed data management system, data was collected and analyzed from two medical institutions: University Hospitals of Basel and Clermont-Ferrand.

Separate REDCap projects were created for each institution, ensuring consistency while allowing for some customization. Both projects utilized forms with a similar structure, but specific fields were adapted to each center’s data collection needs. Notably, the forms were designed with direct data validation in mind. For example, in the targeting information section text entries were restricted to prevent insertion of invalid X, Y, and Z coordinate inputs.

Collaborating physicians diligently filled out these forms. For each patient, completion of 10 distinct forms was required. The process began with the patient information form, capturing demographics and relevant medical history. Subsequent forms documented pre- and post-operative clinical evaluations and medications, available imaging data with accompanying metadata, planned electrode placement parameters, intra-operative microelectrode recordings, details of stimulation procedures and their effects, final electrode positioning, and post-operative screening results. Importantly, many forms were filled out multiple times throughout the process.

In addition to data entry in REDCap, physicians also identified research-relevant images and plans from the neurosurgical planning stations (Brainlab Elements^6^) and exported those. Following data collection by physicians and image data transfer, the system seamlessly integrated the information into the database. This integration was achieved using the two pipelines described in Methods sections 2.4 and 2.5.

The system allowed to successfully collect data from 107 Essential Tremor (ET) and Parkinson’s Disease (PD) patients, including an average of 35 imaging files per patient. These files encompass MRI and CT scans, along with labeled anatomical structures. The data is securely stored and managed within the data management system. An illustrative scenario where database-driven data integration is crucial occurs during the simulation of brain tissue volume activated by specific stimulation parameters. This process involves generating a patient-specific brain tissue model from their MRI scan and inputting the stimulation configuration into the simulation software. The developed database serves as a centralized repository for all necessary information: stimulation parameters can be accessed via querying the ‘Stimulations’ table, while the file path to the patient’s brain image is retrievable from the ‘Files’ table. Upon generation of simulation files, references to them are stored in both the ‘Files’ and ‘Bids’ tables. Furthermore, the system’s true power lies in its ability to facilitate group-level analysis. Its design allows the streamlined selection of all requisite files for the generation of disease-specific anatomical atlases or stimulation maps in a singular, efficient step. Additionally, the system processes and stores files generated during post-processing steps, guaranteeing data integrity and enabling clear traceability throughout the research process. The repository NeuroDataManagementSystem^7^ contains extracted sample REDCap data and image files, together with the BIDS project folder and populated SQLite database resulting from the application of the REDCap2SQLite and Image2BIDS2SQLite pipelines.

## 4 Discussion

Streamlining data capture and management is critical for advancing neuroscience research. Tackling the challenges posed by handling extensive volumes of heterogeneous data collected across multiple sites requires the implementation of a robust data management system. Such a system can bring enhancements in data quality (I), organization (II), analysis (III) and collaboration (IV) among medical and scientific institutions. The system presented in this manuscript offers progress in all of these points. The usage of a web-platform facilitates the transition away from paper or spreadsheet-based data collection methodologies. Data validation functionalities allow to minimize errors associated with manual data entry, ensuring data accuracy and consistency (I). Intuitive web forms require minimal computer proficiency by the user. Furthermore, the compliance to General Data Protection Regulation (GDPR) guarantees secure data sharing between researchers and clinicians, fostering collaborative research efforts (IV). All of these attributes, coupled with its cost-free accessibility, made REDCap the optimal selection for clinical data acquisition (Harris et al., 2009).

Data structure standardization strongly impacts cross-lab collaborations and study reproducibility (Zehl et al., 2016; Zwiers et al., 2022). Considering its expanding adoption within the neuroscience community, we structured our imaging data in accordance with the BIDS standard (Gorgolewski et al., 2016). A customized tool automates the conversion of image files from DICOM to NifTI format and arranges them in a BIDS-compliant structure, taking care of the laborious task of manually naming and saving files. BIDS was initially designed to facilitate the organization of raw imaging data and associated metadata. Nonetheless, judicious usage of the derivatives folder allowed to incorporate additional files generated by analysis pipelines while preserving adherence to the standard. As a result of this implementation, output files deriving from group analysis and transformations across various image spaces can be readily monitored and accessed (II). Recently, BIDS has also been adopted by Lead-DBS (Neudorfer et al., 2023). Lead-DBS is a neuroimaging platform allowing to perform image processing and electrical field simulation operations. Their folder structure also features a first subdivision by subject. However, files are then grouped by processing step rather than by content. Moreover, files belonging to a common anatomical space (e.g. atlas files) are not collected in a group analysis folder but they are included in patient folders. This highlights an alternative perspective: the utilization of BIDS derivatives offers flexibility in organizing files and accommodating various data types within the standard, albeit leading to increased variability in folder structures across different research groups. Consequently, the implementation of additional guidelines for organizing derivatives could prove advantageous.

Group and longitudinal analyses can strongly benefit from the joint power of demographics, clinical and imaging data. Nevertheless, a problem which frequently arises when dealing with heterogeneous data is maintaining the connections between metadata and the data objects to which they refer (Zehl et al., 2016). Recently, a solution for integrating data collected via REDCap with imaging data structured according to BIDS has been presented (Kuplicki et al., 2021). It consists in exporting clinical measures stored in REDCap in tsv files saved in the BIDS folders. However, this solution does not optimally facilitate data retrieval and fails to provide a holistic picture of the collected data. To overcome these limitations, we modeled and implemented an SQLite relational database. Self-developed tools enable the automatic population of database records with data sourced from REDCap and references to imaging files. This approach allows the database to provide a complete overview of the available data and to serve as a unified interface for accessing it. In recent years, data management systems based on a relational database backend have emerged within the neuroscience community (Marcus et al., 2007; Keator et al., 2009; Van Horn and Toga, 2009; Prodanov, 2011; Scott et al., 2011; Das et al., 2012; Book et al., 2013; Muehlboeck et al., 2014; Woodman et al., 2014; Grigis et al., 2017). Nonetheless, a considerable portion of these systems currently lacks compatibility with BIDS-compliant data standards. Furthermore, although these systems primarily focus on improving neuroscience data sharing among researchers, they have not been specifically designed with consideration for specific clinical and research workflows such as inherent to DBS. Consequently, their adoption would have required significant effort in user training and adaptation to DBS-specific use cases and pipelines. The database presented in this manuscript employs a DBS-specific data model, structured to correspond with the various sources of data involved in the DBS procedure. Moreover, a patient-centric approach was followed, as was also done in Neuroinformatics Database providing a platform for storing, analyzing and sharing neuroimaging data [NiDB (Book et al., 2013)] and Longitudinal Online Research and Imaging System [LORIS (Das et al., 2012)], dealing with multi-center, heterogeneous data acquisition, organization and dissemination. This decision was made considering its compatibility with both clinical procedures and analytical frameworks.

By harnessing the synergy between REDCap, BIDS, and SQLite, a data management system fostering heterogeneous data integration and handling for collaborative research purposes has been successfully engineered. In addition, although the database schema is DBS-specific, the pipelines enabling inter-platform communication and the overall framework concept can be easily employed in other neuroscience fields. The adoption of such a system holds the potential to substantially enhance the speed, efficiency, and robustness of research advancements reliant on large-scale data analyses. One of the advantages of utilizing a system comprising three interoperable yet independent components is that the temporary failure of one element does not compromise the functionality of the others. For instance, a temporary unavailability of the SQLite database does not entirely impede data retrieval: clinical data can still be directly exported from REDCap, and imaging data can be retrieved from the BIDS project folder, together with the relevant metadata contained in the JSON sidecar files. Although this would increase the complexity and time required for data integration, it would not completely halt workflows or, more importantly, result in data loss. Nonetheless, this system still constitutes the initial prototype of a comprehensive data management platform. Moving forward, some limitations will require attention and resolution in subsequent developments. The database is presently equipped with a suite of Python functions facilitating straightforward data query, insertion, update, and deletion. An initial enhancement would involve integrating a graphical user interface (GUI). This augmentation would enable researchers lacking coding proficiency to query data and visualize the query results, thereby enhancing the usability and user-friendliness of the system. A subsequent step would entail integrating statistical analyses or processing procedures with the system. In this way such analyses could be directly executed from the GUI rather than using external scripts. Finally, analyses outcomes data are currently accessible exclusively to authorized users within our institution. Implementing web-based access to the database would facilitate retrieval of DBS research output data by members of the scientific community. This would thus increase accessibility and utilization of such data.

## 5 Conclusion

Modeling and managing workflows play a crucial role in both present and future neuroscience endeavors. This is especially noticeable in light of big data continuous evolution and increasing collaborations in the scientific community. This work demonstrates the successful integration of a translational tool for streamlining data collection and organization between clinics and research institutes in the field of Deep Brain Stimulation. The proposed framework captures both standardized imaging data and comprehensive patient-metadata within a unified system. The system has demonstrably achieved successful validation through data collection and analysis from two medical institutions. It offers efficient storage and management of large-scale clinical and imaging data, while simultaneously simplifying data retrieval for group-level analysis. This approach enables researchers in the field of DBS to leverage the richness of diverse data types, potentially leading to improved clinical decision-making and ultimately, better patient outcomes.

## Data Availability

The original contributions presented in the study are included in the article/Supplementary material, further inquiries can be directed to the corresponding author.

## Footnotes

1
https://www.sqlite.org/

2
https://www.comsol.com/

3
https://github.com/IM2Neuroing/REDCap2SQLite/releases/tag/v1.0.0

4
https://www.dicomstandard.org/

5
https://github.com/IM2Neuroing/Image2BIDS2SQLite.git

6
https://www.brainlab.com/radiosurgery-products/elements/

7
https://github.com/IM2Neuroing/NeuroDataManagementSystem.git
