# Supplementary material for: Optimizing neuroscience data management by combining REDCap, BIDS and SQLite: a case study in Deep Brain Stimulation
Source: Front Neuroinform. 2024 Sep 5;18:1435971. doi: 10.3389/fninf.2024.1435971 (PMC11410584; doi:10.3389/fninf.2024.1435971)
Supplement: Supplementary file 1 [file Data_Sheet_1.docx]

Supplementary Material

The document contains pictures of sample REDCap project dashboards and data collection forms. The patients’ records shown in the pictures do not belong to any real patient. They have been populated with fictitious data for demonstration purposes.


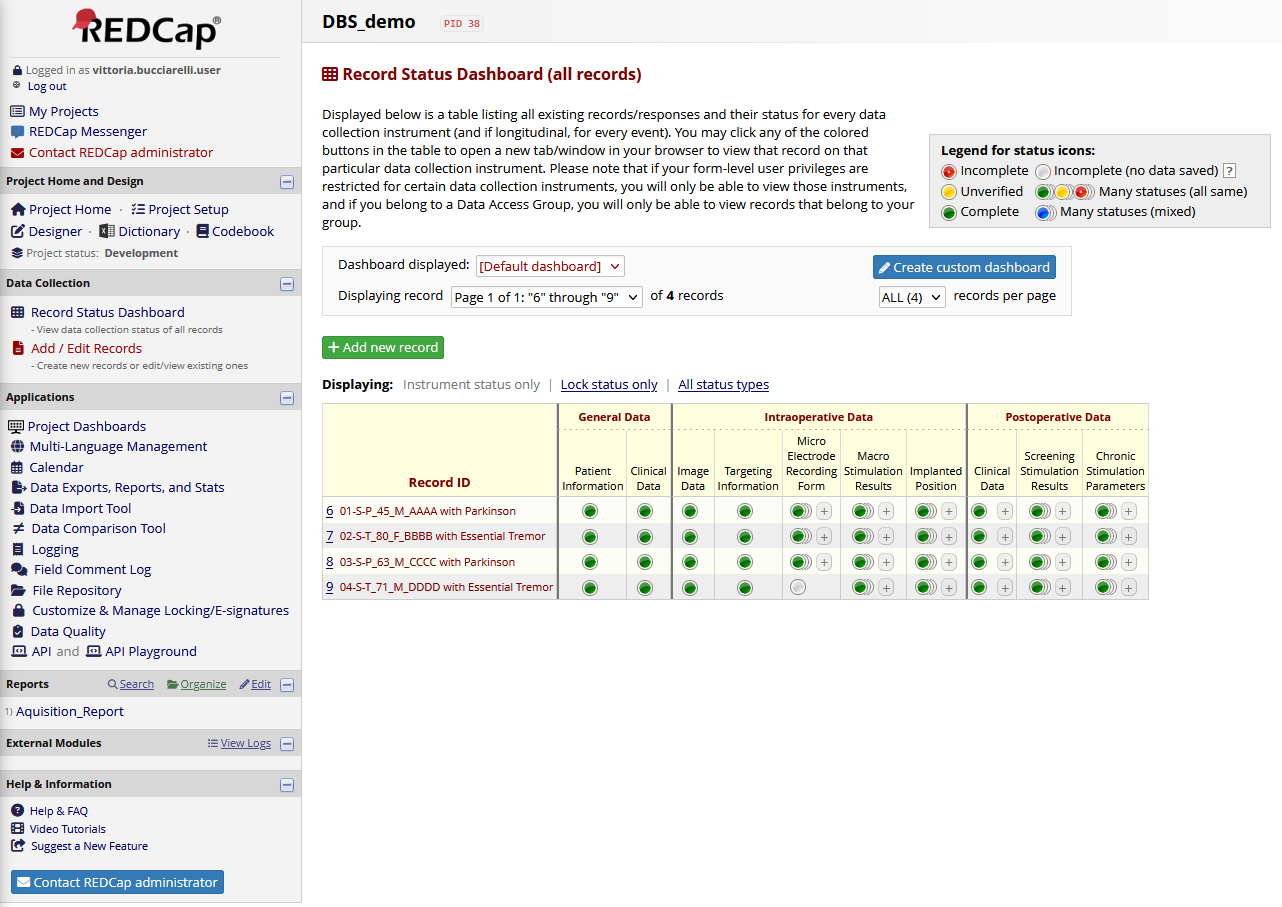


Figure S1. REDCap demo project dashboard. REDCap project dashboard shows the patients whose data has been collected in the context of the project. For each patient it is possible to see which data has been collected and the color of the icons indicates the status of each data form. This allows to immediately visualize if the forms are complete or incomplete.


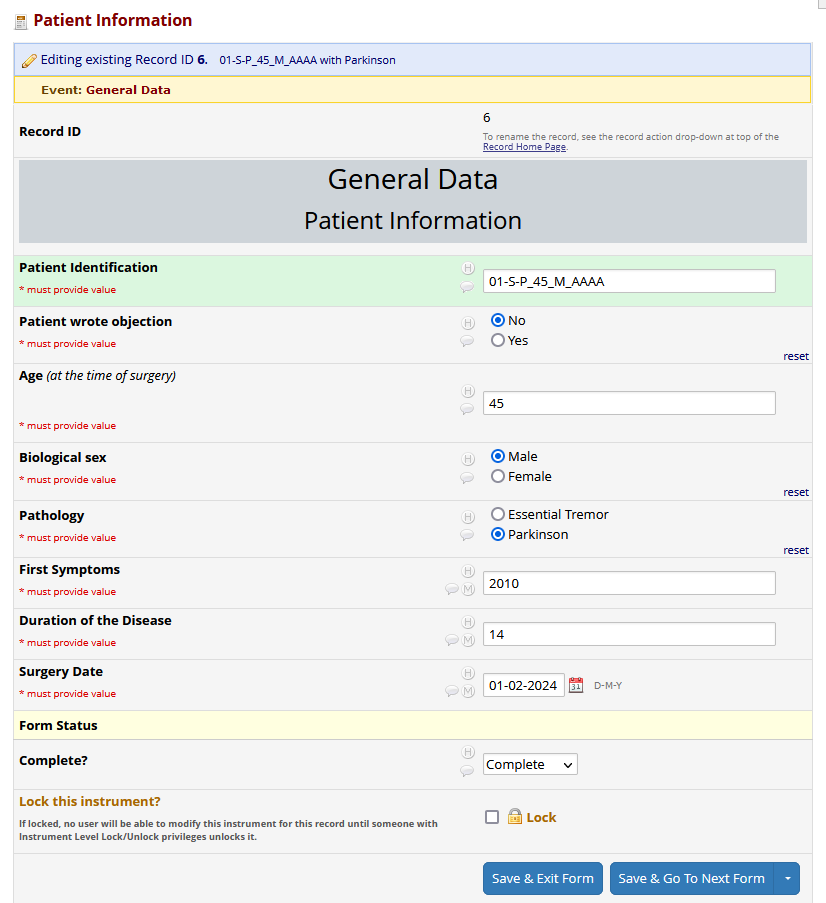


Figure S2. REDCap demo patient information form. REDCap allows to create forms where data can be inserted in text boxes (for strings, numbers, dates), note boxes (for long text), drop-down lists, checkboxes, sliders. It also allows to upload files, to flag some fields as mandatory and to have fields calculated on other fields’ values.


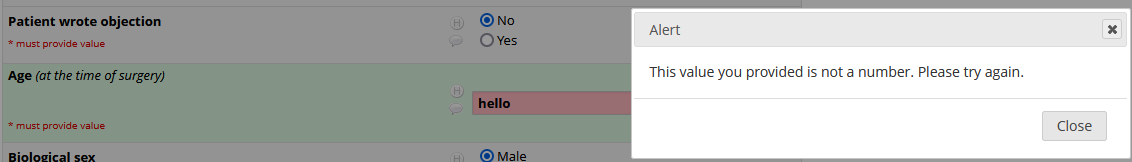


Figure S3. REDCap data validation. REDCap allows to perform data validation. In the presented example the user is notified that the field “Age” only accepts numeric values.


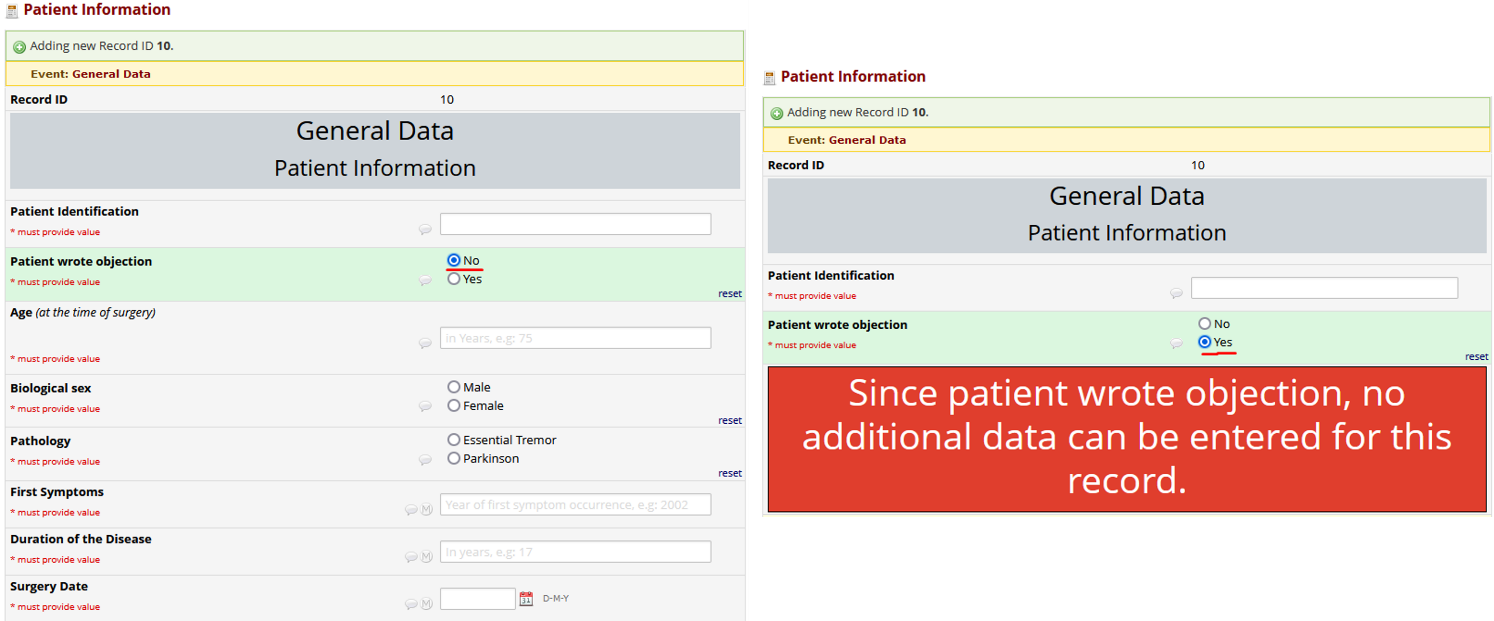


Figure S4. REDCap branching logic. Some fields can be made visible only when other fields meet certain conditions. For example if the patient provided an objection (on the right) no additional data can be entered.


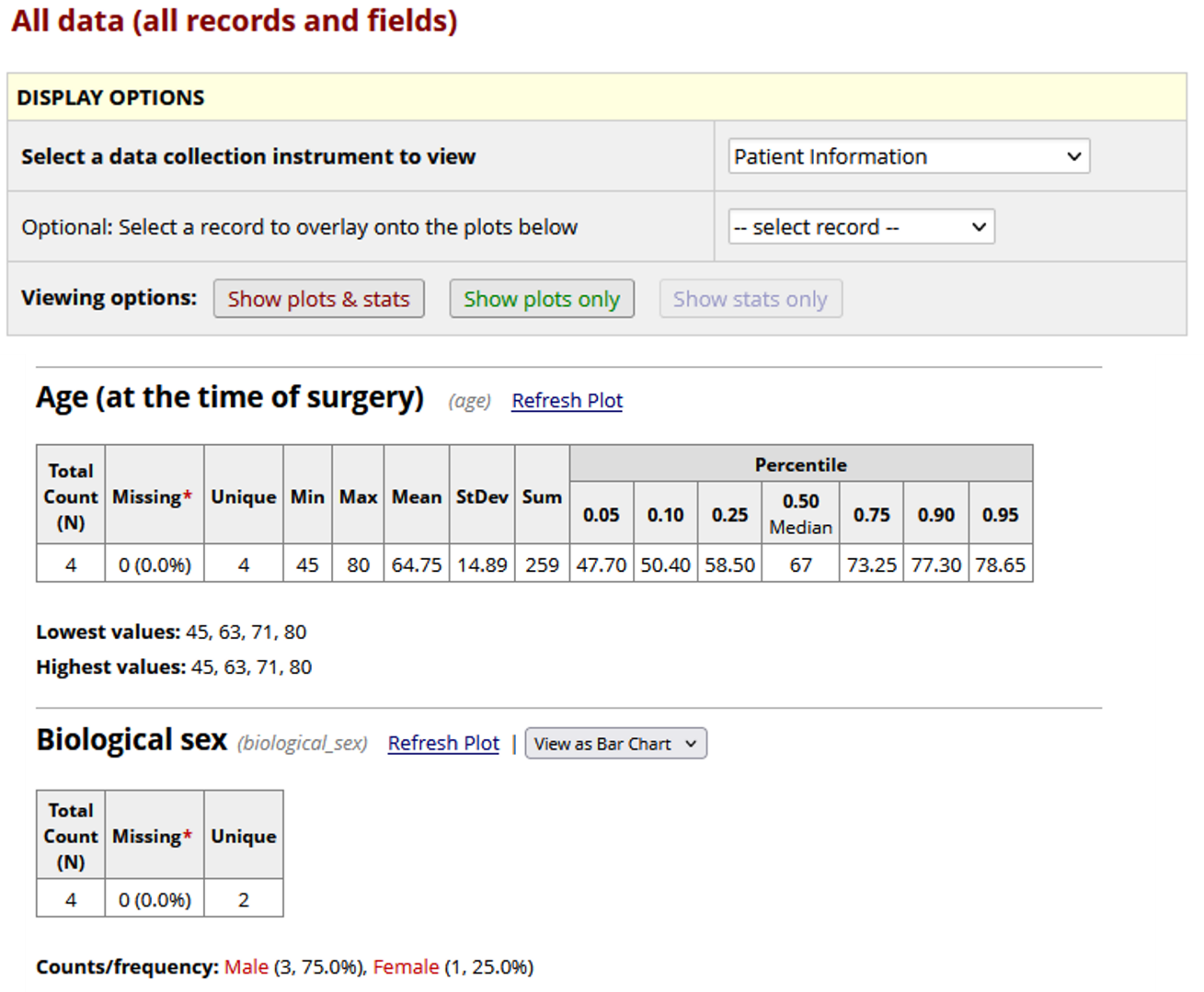


Figure S5. Data statistics. It is possible to visualize the collected data statistics, either in table format or plot.


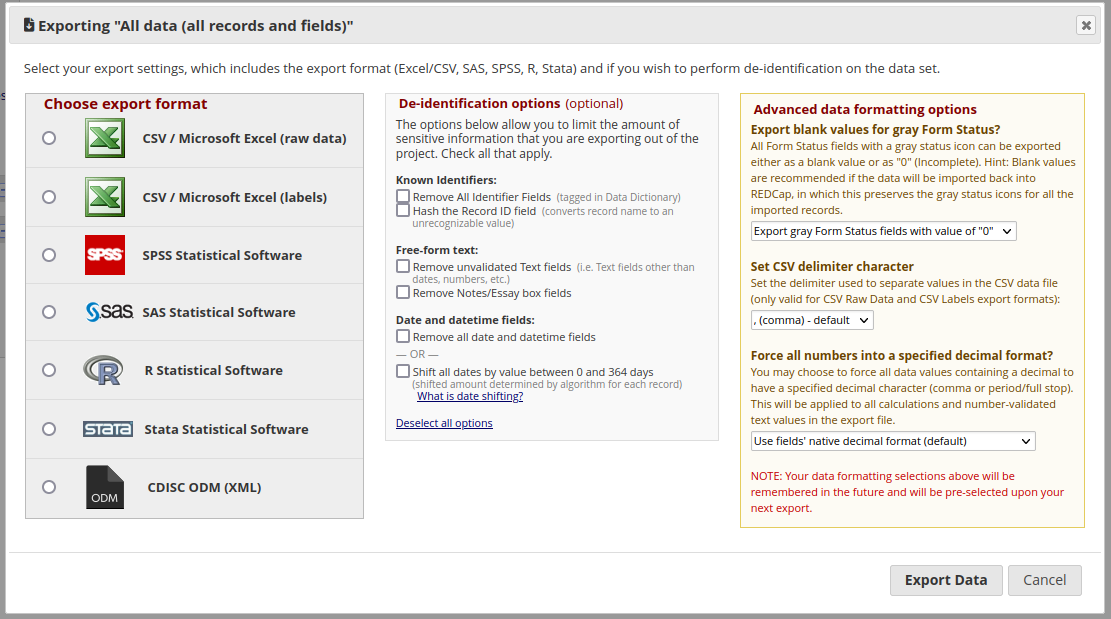


Figure S6. Data export. Data can be easily exported in formats compatible with Excel, SPSS, SAS, R, Stata, CDISC ODM.
